# Cryptic diversity in *Andrognathuscorticarius* Cope, 1869 and description of a new *Andrognathus* species from New Mexico (Diplopoda, Platydesmida, Andrognathidae)

**DOI:** 10.3897/zookeys.786.27631

**Published:** 2018-09-25

**Authors:** Patricia L. Shorter, Derek A. Hennen, Paul E. Marek

**Affiliations:** 1 Virginia Polytechnic Institute and State University, Department of Entomology, Price Hall, 170 Drillfield Drive, Blacksburg, Virginia, USA Virginia Polytechnic Institute and State University Blacksburg United States of America; 2 Louisiana State University, Department of Entomology & School of Renewable Natural Resources, Life Sciences Building, Forestry Lane, Baton Rouge, Louisiana, USA Louisiana State University Baton Rouge United States of America

**Keywords:** COI, cryptic species complex, distribution map, millipede, neotype, phylogeography, scanning electron microscopy, taxonomy

## Abstract

*Andrognathus* is a genus of small, thin-bodied millipedes found in deciduous forests of North America. Poorly understood, these organisms inhabit decaying wood and have morphologically conserved and difficult-to-identify sexual characters that have limited study historically. Recent use of scanning electron microscopy has uncovered variation in male genitalia that was previously unknown in the genus. The distribution of *Andrognathus* and the extent of this variability across the continent, however, were undocumented, and a wealth of natural history collections remained uncatalogued. Here a new species of *Andrognathus* is described from New Mexico, *Andrognathusgrubbsi***sp. n.**, natural history collections are utilized to create a comprehensive map of the genus, and a neotype established for the type species, *Andrognathuscorticarius* Cope, 1869. Analysis of the cytochrome oxidase I gene (COI) for *A.corticarius* was completed for the type series and individuals across the species distribution, but little variation was found. *Andrognathusgrubbsi***sp. n.** joins *A.corticarius* and *A.hoffmani* Shear & Marek, 2009 as the only members of the genus.

## Introduction

**Figure 1.**
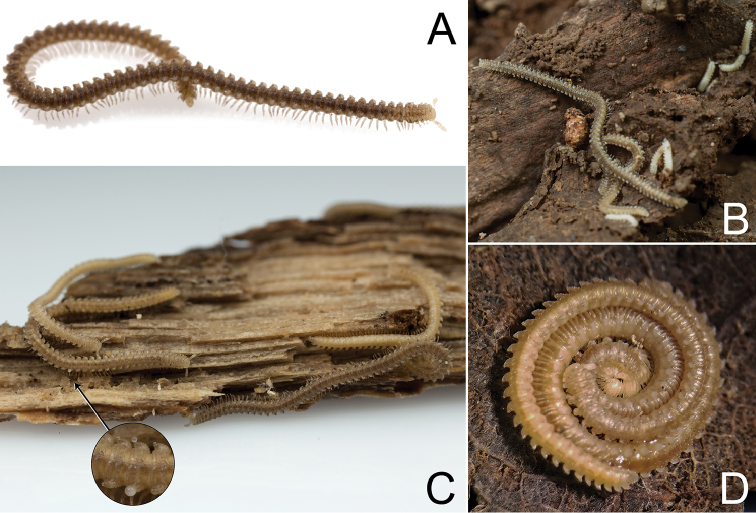
*Andrognathuscorticarius* Cope, 1869 from Stadium Woods, Montgomery County, Virginia **(A–C)** Pulaski County, Virginia **(D)**. **A** Adult male, dorsolateral view, approximate length 24 mm (VTEC catalog #MPE01962) **B** Adults and juveniles *in situ*. Aggregated individuals were found inside a decaying hardwood log **C** Adult aggregation. Inset shows the chemical secretions on the ozopores on the bottom left and bottom right individuals **D** Male and female coiled around eggs.

**Figure 2.**
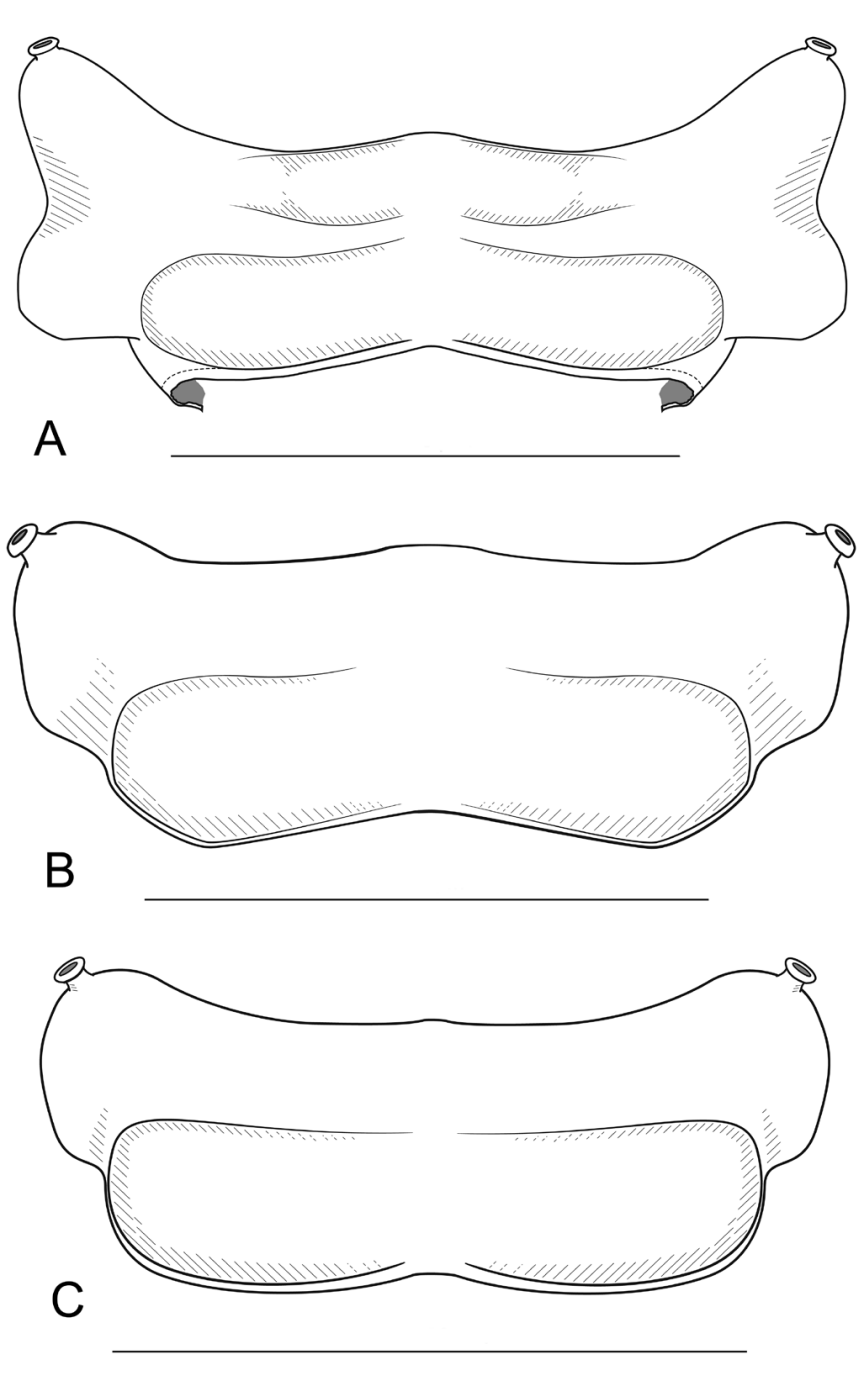
*Andrognathus* ring 5, dorsal view. **A***Andrognathuscorticarius***B***Andrognathushoffmani***C***Andrognathusgrubbsi* sp. n. Scale bars: 0.5 mm (**A, C**); 0.4 mm (**B**).

**Figure 3.**
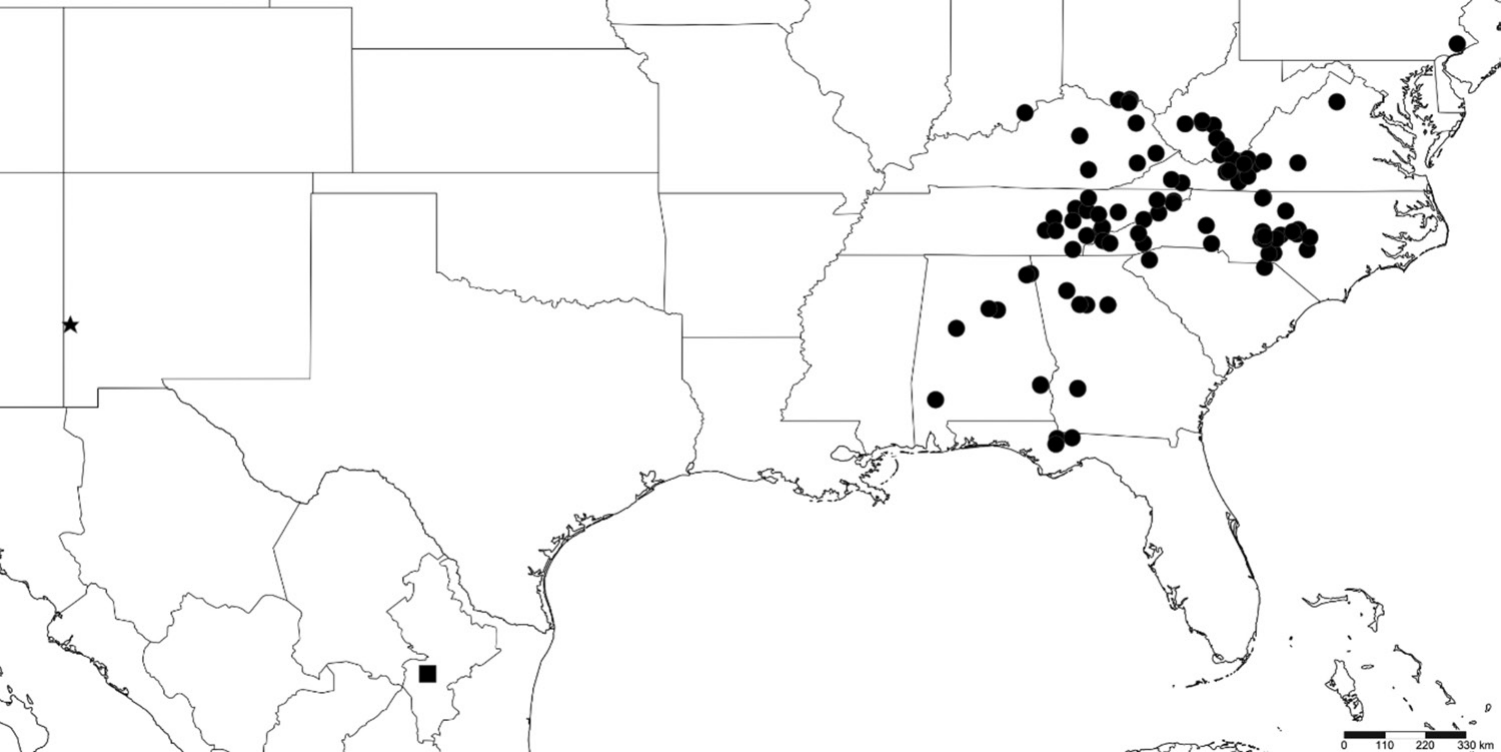
Distribution map of *Andrognathus*. Dots: *Andrognathuscorticarius*, square: *Andrognathushoffmani*, star: *Andrognathusgrubbsi* sp. n. The species *A.corticarius* is reported from Ohio and Pennsylvania for the first time, and the genus is reported from New Mexico for the first time.

*Andrognathuscorticarius* Cope, 1869 is a small-bodied platydesmidan millipede broadly distributed in mixed mesophytic deciduous forests in the eastern United States (Figure 1A). It is commonly found in aggregations of adults and juveniles beneath bark (Figure 1B, C) (hence the specific name *corticarius*). A diagnostic feature of the species is the shape of the paranota of the fifth body ring, which are bilobed with the ozopores elevated on stalks and directed anteriorly (Figure 2A) (Cook and Loomis 1928). The known range of *A.corticarius* extends from the panhandle of Florida, north into southern Indiana and Pennsylvania (Figure 3). The species is a member of the subterclass Colobognatha, a group that includes millipedes with mouthparts generally reduced in size, and eight leg-pairs anterior to the primitive leg-like gonopods (9 and 10) that have a plesiomorphic complement of six podomeres (Latzel 1884; Hoffman 1980). Colobognatha encompass taxa with superlative leg counts (e.g., *Illacmeplenipes* Cook & Loomis, 1928 with 750 and *Siphonophoramillepeda* Loomis, 1934 with 742), and are euanamorphic, meaning they add leg-pairs and segments throughout their lifespan for an indeterminate amount of time (Enghoff et al. 1993). Colobognatha includes four orders: Polyzoniida, Platydesmida, Siphonocryptida, and Siphonophorida (Golovatch et al. 2015), which are generally differentiated by the shape of the head and variable fusion of the pleura and terga (Hoffman 1980). The presence of ocelli and antennal shape are other primary characters that differentiate these orders. While colobognaths are often collected, and a considerable amount of material in natural history collections exists, they are poorly understood due to their small size and apparently invariant gonopods. As a result, the Colobognatha are notorious as a challenging group (Read and Enghoff 2009), and the taxon’s last global taxonomic synthesis was by Attems (1951).

The order Platydesmida is differentiated from other Colobognatha by having chewing mouthparts, the trunk ring pleurites fused with the tergites, and a gnathochilarium composed of a plesiomorphic five sclerites (Enghoff et al. 2015). Members of the order possess chemical defenses containing heterocyclic nitrogen-containing alkaloids and some members display paternal care (Gardner 1975; Shear 2015). Some species of platydesmidans, such as the andrognathid genera *Brachycybe* Wood, 1864 (Gardner 1975) and *Pseudodesmus* Pocock, 1887 (Lewis 1984), aggregate in a stellate pattern, where the cephalic ends of multiple individuals point inward toward a central hub and telsons radiate outward in a star or spoke-like configuration (termed a pinwheel). *Andrognathuscorticarius* has not been found in these formations. The families Andrognathidae and Platydesmidae are differentiated solely by the morphological trait of narrow sternites that make the coxae appear to be nearly contiguous in andrognathids (Cook and Loomis 1928; Enghoff et al. 2015). However, proximity of coxae may be a homoplasy, and based on a recent phylogenomic analysis of the Diplopoda, the genera *Platydesmus* and *Brachycybe* are more closely related to one another than either is to *Andrognathus* thereby indicating non-monophyly of the family Andrognathidae (Rodriguez et al. 2018). Andrognathidae currently has 12 genera with a primarily Holarctic distribution, but two genera are found in Southeast Asia: *Brachycybe* Wood, 1864 and *Pseudodesmus* Pocock, 1887 (Hoffman 1980; Enghoff et al. 2015). The family is divided into three subfamilies: Dolisteninae Latzel, 1884; Bazillozoniinae Verhoeff, 1935; and Andrognathinae Cope, 1869 that contains *Andrognathus* as its sole genus (Hoffman 1980).

*Andrognathuscorticarius* is the type species for the genus and was discovered by Edward Drinker Cope in Virginia more than a century ago. Cope’s fieldwork was focused on cave species, primarily fossil vertebrates, but he also collected myriapods. Cope listed the type locality of *A.corticarius* only as “Montgomery County, Virginia” (Cope 1869: 182).

Unfortunately, the type material of *A.corticarius* has not been located and is presumed lost (Hoffman 1999; see also below Materials and Methods section). For 138 years, the genus contained only *A.corticarius*; however a series of andrognathids that possessed the characteristic lobed paranota of the fifth body ring were discovered in Nuevo Leon, Mexico by Casey Richart in 2007. These specimens were subsequently described as the species *Andrognathushoffmani* Shear & Marek, 2009. In the same paper, the authors indicated that a cryptic species complex may exist in *A.corticarius*, stating that it deserves a more detailed examination (Shear and Marek 2009). However, the lack of type material of *A.corticarius* has meant that the identity of the species has remained uncertain.

The small body size of *A.corticarius* and its apparent uniformity at low optical magnification may underestimate the diversity of the species. Here we use high-magnification imaging to document the range of genitalic variation in *A.corticarius* and suggest that the species is a cryptic species complex. To stabilize the taxonomy of the group we designate a neotype for *A.corticarius* and describe a new *Andrognathus* species from New Mexico, United States.

## Materials and methods

*Material examined.* Natural history collections and newly collected specimens of *Andrognathus* were used for this study. Material collected in the field (2005–2017) was brought back to the lab alive for DNA extraction and specimen preparation. Individuals were found by flipping and breaking apart decaying hardwood logs and examining the surfaces of large fallen branches in mesic forest habitats, such as areas where xystodesmid millipedes are found (Means et al. 2015). In contrast, some individuals were occasionally found in dry habitats under pine logs. *Andrognathuscorticarius* individuals appear to burrow into dead organic matter and humus, and are often found inside decaying wood and beneath the bark. They are not typically beneath leaves as is commonly observed with xystodesmid millipedes (Means et al. 2015). Decaying logs that were just beginning to lose bark and soften due to decomposition were ideal habitats. Collected specimens were labelled with locally unique identification (LUID) codes beginning with the prefix “MPE-”, and were deposited in Virginia Tech Insect Collection (abbreviation VTEC, https://www.collection.ento.vt.edu) These data are available for download from VTechData (https://data.lib.vt.edu/collections/44558d39p, https://doi.org/10.7294/kbtb-8v48, Suppl. material 1).

Other material for this study comprised specimens deposited in the North Carolina State Museum (NCSM) and the Virginia Museum of Natural History (VMNH) (Suppl. material 1). These collections were chosen as they are the largest repositories of *Andrognathus* specimens. These specimens were given labels with the LUID code, “AND-”. The collection labels of these specimens were georeferenced with GEOLocate to recover the geographical coordinates of the occurrence (Rios and Bart 2010). Elevation was estimated using Google Earth (http://www.earth.google.com). As it documented the northernmost locality of the genus *Andrognathus*, we included an observation in Pennsylvania from the citizen science observation website BugGuide (BugGuide.net 2016) and confirmed the validity of the record with the observer; these specimens were not collected. A distribution map for the species utilizing specimens collected for this study, along with georeferenced localities from the literature (Gardner 1975, Shear and Marek 2009, Shelley 1978, Shelley 2000) (Suppl. material 2) was generated with the online GIS software Simplemappr (Shorthouse 2010) (Figure 3). These data are also available for download from VTechData (https://data.lib.vt.edu/collections/44558d39p, https://doi.org/10.7294/kbtb-8v48).

The Academy of Natural Sciences of Drexel University (ANSP) in Philadelphia, where Cope traditionally deposited his material, was contacted to determine if any type material was present in their holdings. The type database of the Museum of Comparative Zoology at Harvard (MCZ), a second repository of Cope material, was searched online to determine if the material was deposited there (MCZBase 2017). No andrognathid specimens assignable to the original types of Cope were present in either of the collections. Hoffman (1999) speculated that the type locality of *A.corticarius* is Yellow Sulfur Springs (Montgomery County, Virginia), a popular resort in the late 19th century. Fourteen male and seven female specimens of *A.corticarius* were collected at Yellow Sulfur Springs in 2016.

*Morphological analysis and imaging.* Seventeen well-preserved males of *A.corticarius* (Table 1) were selected for SEM imaging including individuals from: Liberty County, Florida (southernmost known point); Dekalb County, Alabama (westernmost); Kanawha County, West Virginia (northernmost); and Campbell County, Virginia (easternmost). Only females were available from other peripheral portions of the species range.

**Table 1.** Measurements (in mm) Anatomical details for *A.corticarius* individuals from the edges of the geographic distribution, and for the neotype (*). Cells with a hyphen (-) indicate specimens lacking the feature due to destruction for genetic analysis. (^†^) Ring count does not include the two apodous rings (a) and the telson (T).

Code	State	Sex	Ring Count^†^	Leg Count	BL	HW	CW	W1
MPE01991	OH	M	–	–	–	0.4	0.5	0.6
MPE01942*	VA	M	57	210	17.9	0.5	0.6	0.8
AND0024	VA	M	34	118	8.2	0.4	0.5	0.8
AND0001	NC	M	45	162	13	0.4	0.6	0.8
AND0031	AL	M	34	118	6.3	0.4	0.5	0.7
AND0038	FL	M	35	122	8.2	0.4	0.6	0.7
MPE01986	OH	F	–	–	–	0.4	0.6	0.8
AND0049	VA	F	41	146	9.5	0.4	0.6	0.8
AND0006	NC	F	43	154	9.8	0.4	0.6	0.8
AND0028	AL	F	55	202	15.9	0.4	0.6	0.8
AND0003	FL	F	51	186	13.8	0.5	0.6	0.8

For morphological analysis, the gonopod-bearing body ring was mounted ventral side up on a 12.7 mm aluminum SEM stub using carbon tape (Pellco, California). The stubs were coated with 20 nm of platinum and palladium with a Leica EM ACE 600 high vacuum coater, and imaged on a FEI Quanta 600 FEG environmental SEM. Micrographs were taken of the gonopod-bearing ring as a whole and of the sixth podomere process of the anterior (A6) and posterior (P6) gonopods. For specimens collected in New Mexico, a male and a female were imaged.

*DNA extraction and genetic analysis.* Fresh material of recently collected individuals was preserved for genetic analysis. The middle third of the millipede trunk was excised and stored in RNALater at -80 °C, and the head, anterior and posterior rings, and gonopods were retained as voucher specimens in 70% isopropanol. The DNA of three individuals from a locality, selected to examine within site nucleotide variation, was extracted and purified using a DNeasy Blood & Tissue Kit (Qiagen, Valencia, California). A region of the cytochrome oxidase I gene (COI) was amplified with polymerase chain reaction (PCR) using the primers LCO1490 and HCO2198 (Folmer et al 1994; Hebert et. al. 2003) according to the methods described in Marek et al. (2012). Amplified DNA was confirmed with electrophoresis on a 10% agarose gel, and raw PCR amplicons were cleaned, quantified, and Sanger-sequenced at the University of Arizona Genomics Core on an ABI 3100 capillary DNA sequencer. Raw chromatograms were analyzed in the Mesquite module Chromaseq version 1.2 using phred and phrap for nucleotide base-calling, trimming, and quality control (Maddison and Maddison 2010; Ewing et al. 1998). The COI sequences were translated into amino acids, and aligned by eye. Mean uncorrected pairwise distances were calculated using Mesquite. A pairwise distance matrix for these data are available for download from VTechData (https://data.lib.vt.edu/collections/44558d39p, https://doi.org/10.7294/kbtb-8v48, Suppl. material 3).

## Results

Specimens included in this study provided 55 localities for *Andrognathus* (including 36 from previously undocumented localities) and resulted in the first comprehensive distribution map of the genus (Figure 3). A total of 274 specimens was examined: 185 individuals (64 males, 83 females, 38 juveniles) were from the NCSM and VMNH, and 89 (45 males, 30 females, 14 juveniles) were newly collected specimens with genomic DNA preserved in the VTEC.

Due to the loss of the holotype and type material of *A.corticarius*, a male individual collected at the presumed type locality in Yellow Sulfur Springs was designated as a neotype. This is listed below in the material examined section. Analysis of the somatic and gonopodal morphology resulted in the delimitation of *A.grubbsi* sp. n. as its own species, and revision of the genus is presented below in the Taxonomy section.

### *Andrognathus* taxonomy

#### Order Platydesmida Cook, 1895

##### Family Andrognathidae Cope, 1869

###### Subfamily Andrognathinae Cope, 1869

####### Genus Andrognathus

Taxon classificationAnimaliaPlatydesmidaAndrognathidae

Cope, 1869

######## Type species.

*Andrognathuscorticarius* Cope, 1869: 182; Gardner 1975: 15, figs 1, 5–7; Hoffman 1999: 180; Shear and Marek 2009: 157, figs 1–10.

######## Other species included.

*Andrognathuscorticarius* Cope, 1869, *Andrognathushoffmani* Shear & Marek, 2009, *Andrognathusgrubbsi* sp. n.

######## Genus diagnosis.

**Figure 4.**
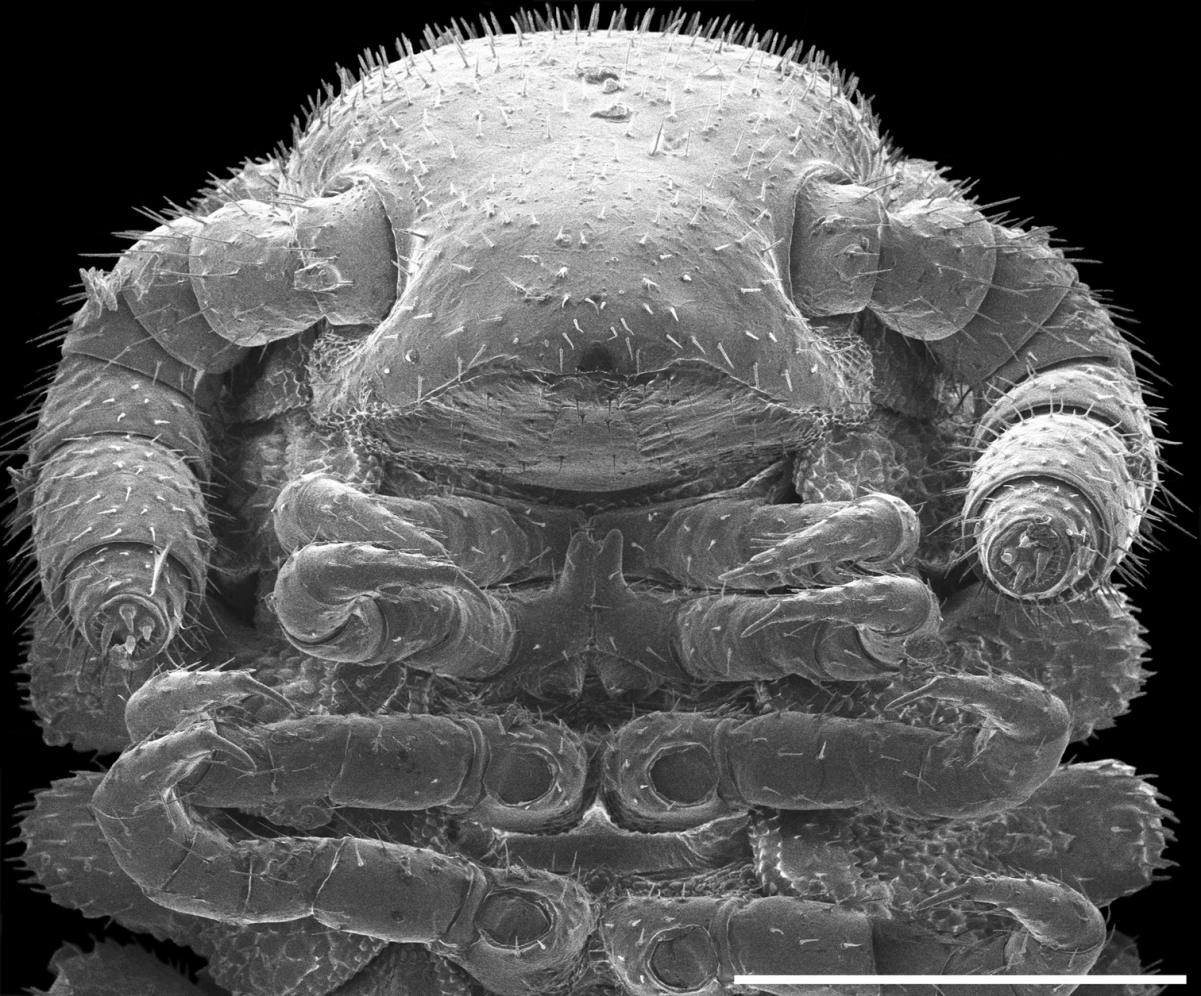
*Andrognathuscorticarius* head and anterior body rings, ventral view. Scale bar: 0.3 mm.

**Figure 5.**
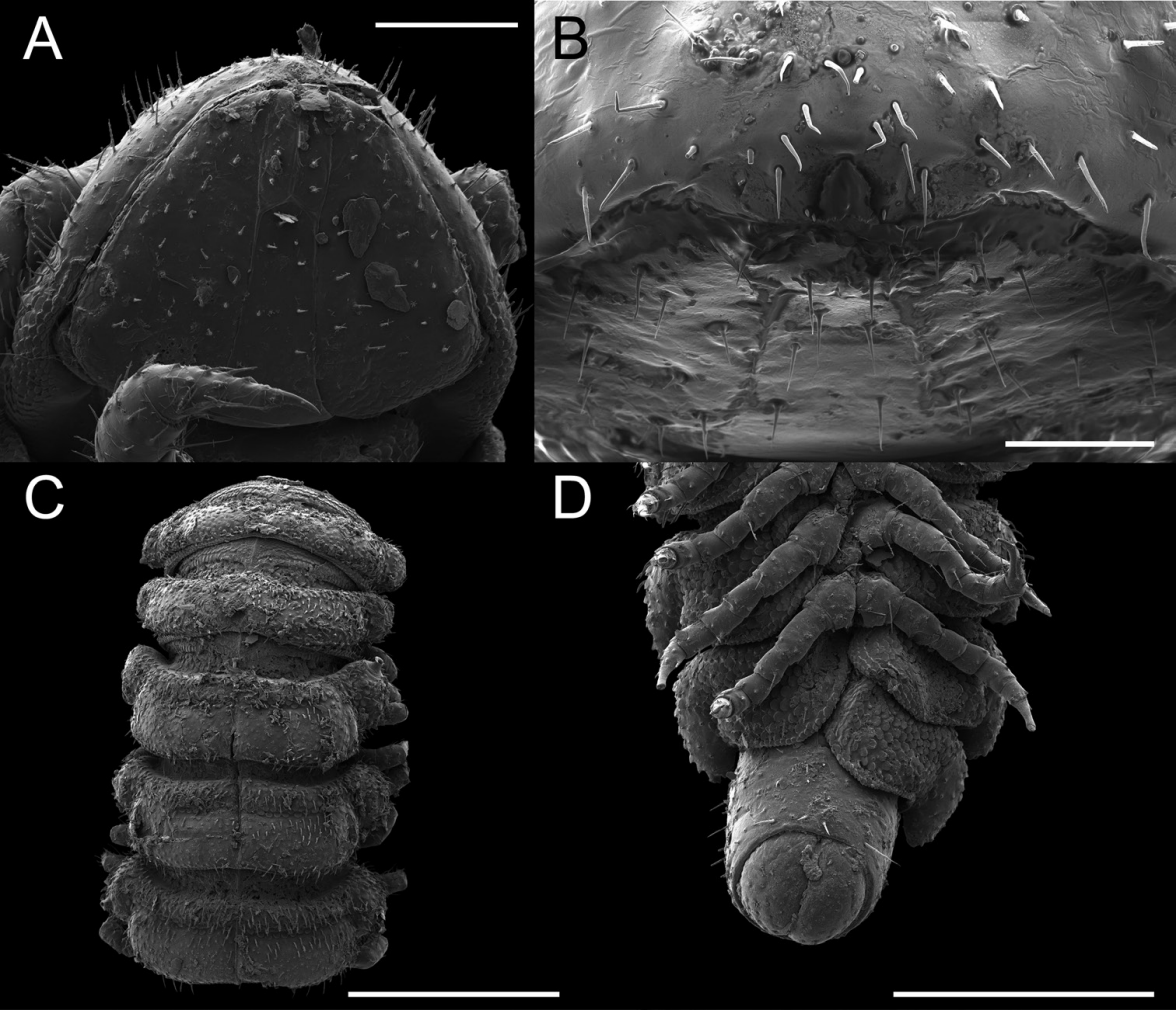
*Andrognathusgrubbsi* sp. n., female paratype, (VTEC Catalog #AND0045). **A** Head, ventral view **B** Tip of labrum **C** Dorsal view of rings 3–7 **D** Posterior body rings, ventral view. Scale bars: 0.1 mm (**A**), 0.05 mm (**B**), 0.5 mm (**C**), 0.4 mm (**D**).

**Figure 6.**
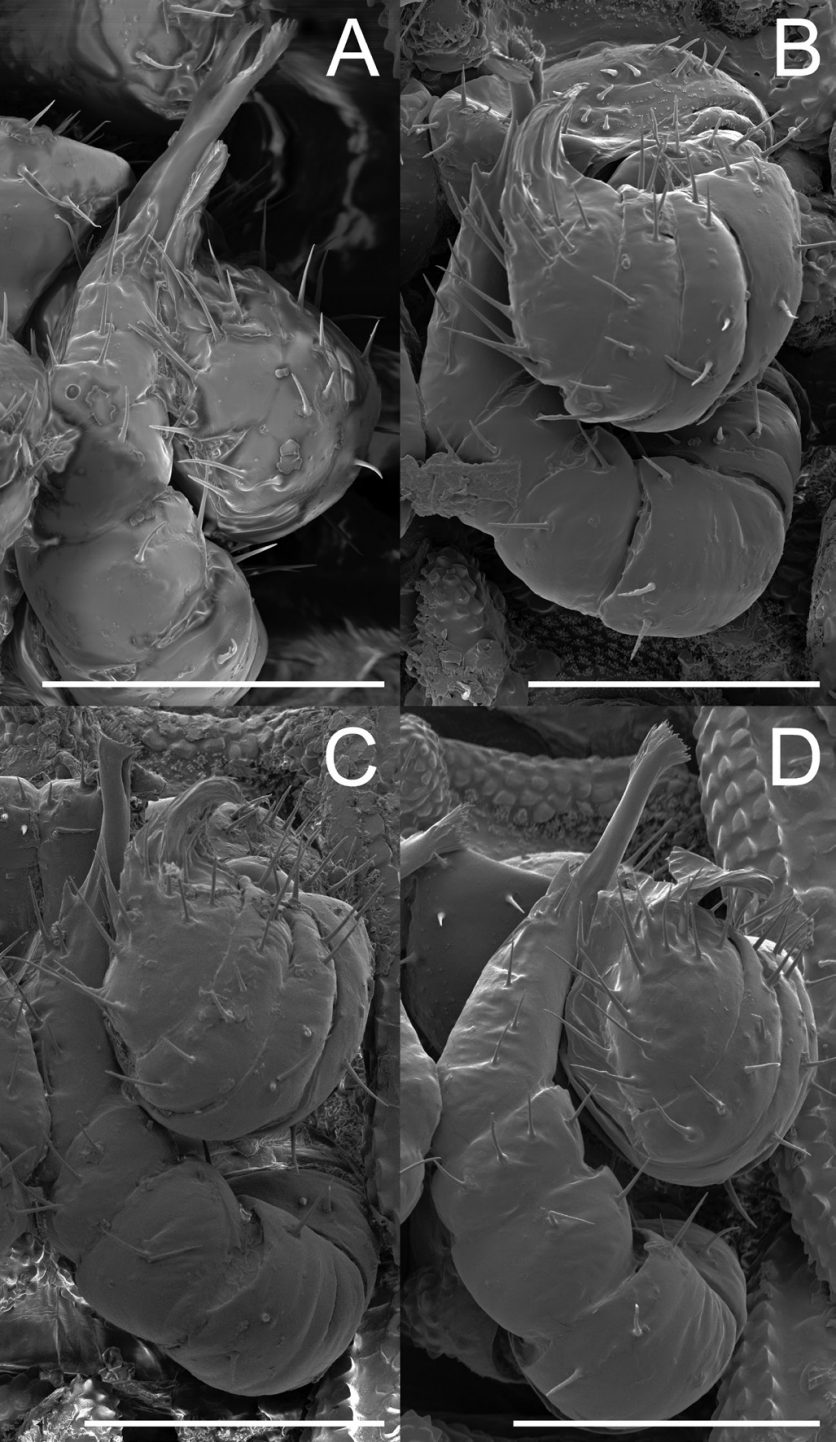
Gonopod variation within *Andrognathuscorticarius*, ventral view, left gonopods. **A** Scioto Co., Ohio (view of the mirrored right gonopod, due to damage to the left gonopod) **B** Raleigh Co., West Virginia **C** Neotype, Montgomery Co., Virginia (Christiansburg) **D** Montgomery Co., Virginia (Blacksburg). Scale bars: 0.1 mm.

**Figure 7.**
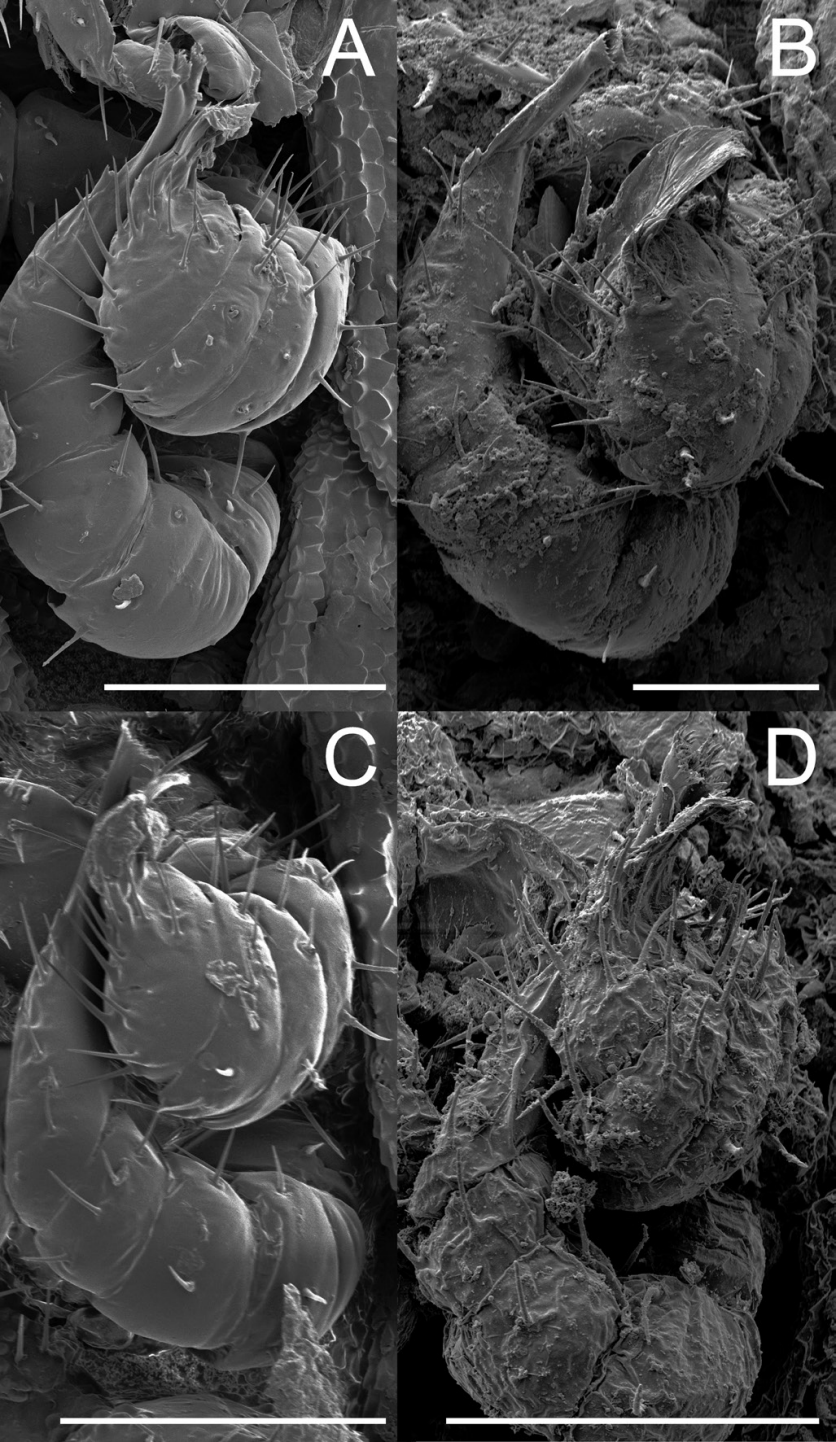
Gonopod variation within *Andrognathuscorticarius*, ventral view, left gonopods. **A** Pulaski Co., Virginia **B** Campbell Co., Virginia **C** Carroll Co., Virginia **D** Madison Co., North Carolina. Scale bars: 0.1 mm (**A, C, D**), 0.05 mm (**B**).

**Figure 8.**
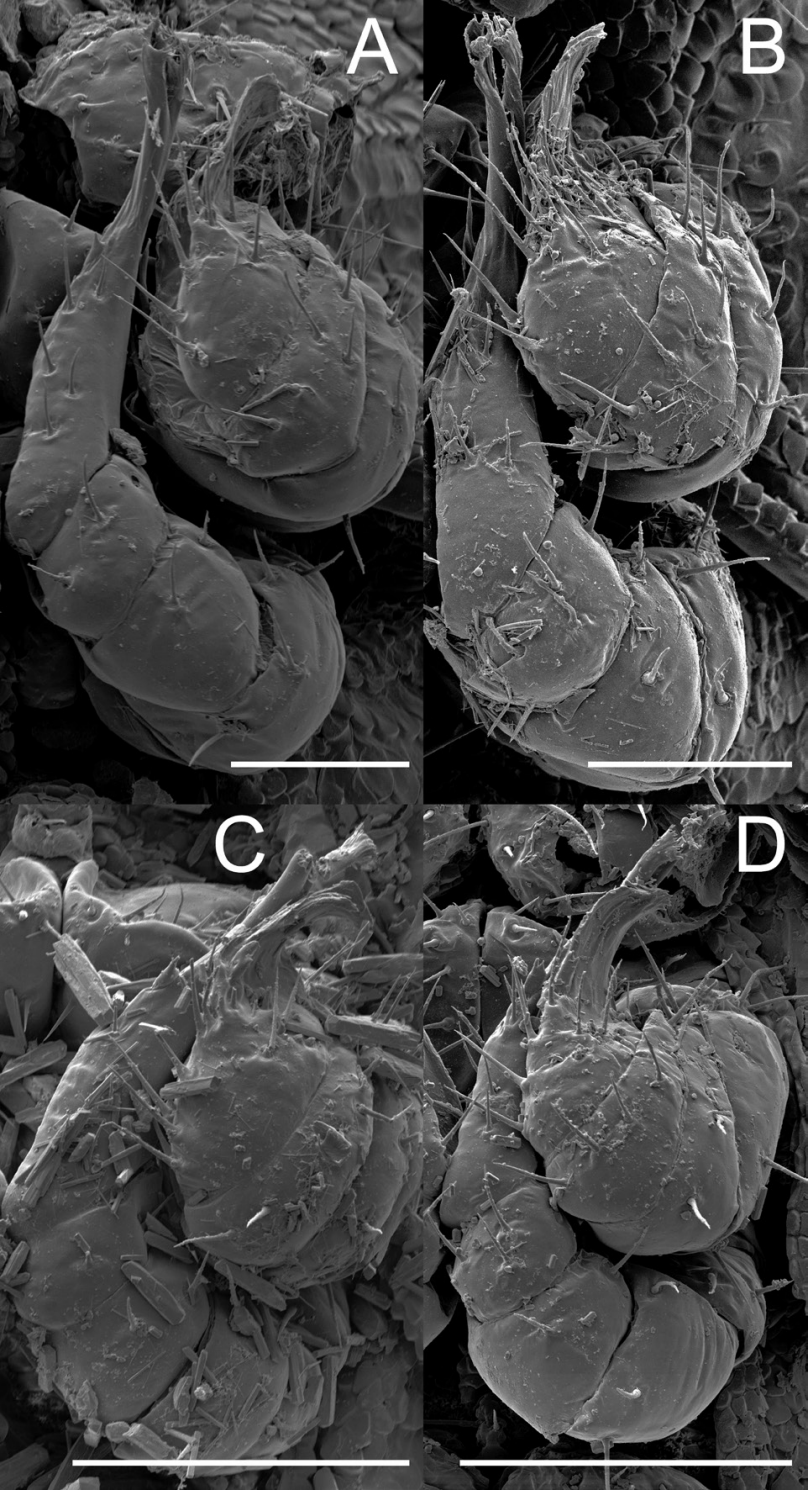
Gonopod variation within *Andrognathuscorticarius*, ventral view, left gonopods. **A** Lee Co., North Carolina **B** Chesterfield Co., South Carolina **C** DeKalb Co., Alabama **D** Liberty Co., Florida. Scale bars: 0.05 mm (**A, B**), 0. 1 mm (**C, D**).

**Figure 9.**
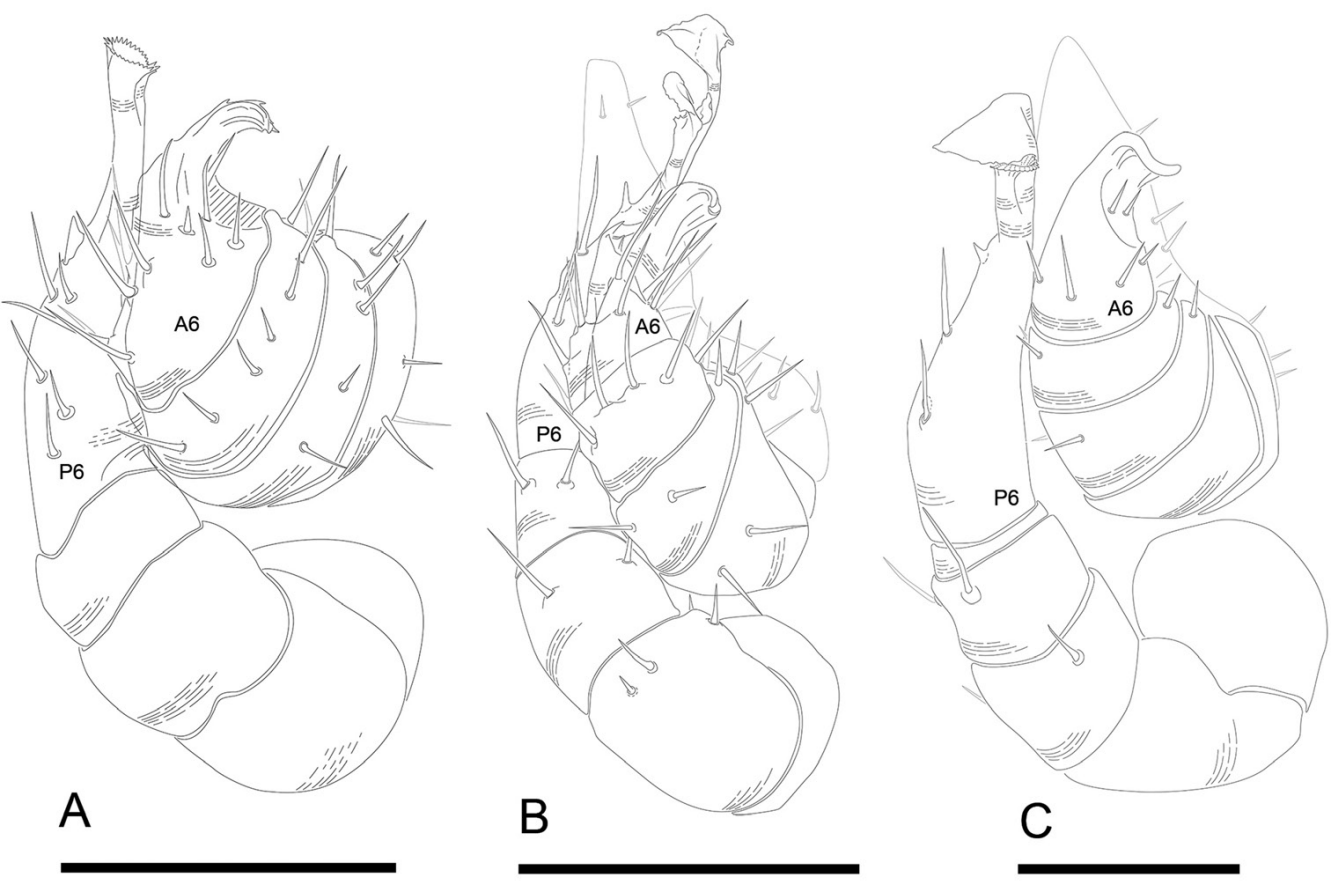
*Andrognathus* gonopods, ventral view of left gonopods. **A***Andrognathuscorticarius* (podomeres A1, A2 missing) **B***Andrognathushoffmani***C***Andrognathusgrubbsi* sp. n. A6: anterior gonopod podomere 6. P6: posterior gonopod podomere 6. Scale bars: 0.1 mm(**A, B**), 0.05 mm (**C**).

Adult *Andrognathus* differ from other andrognathid genera based on the following:

Exoskeleton: Adults with 45–70 rings. Individuals long (11 to 27 mm) and thin, less than one mm wide, with short paranota, not covering legs (as in *Brachycybe*). Color varies from cream to dark brown (Figure 1), with paranota lighter, not pink as in *Gosodesmus* Chamberlin, 1922, *Ischnocybe* Cook & Loomis, 1928, *Brachycybe*. Entire body pilose, particularly head and antennae. Head rounded, pear-shaped, nearly truncate, eyeless (Figure 4). Antennae enlarged apically, slightly elbowed at fourth antennomere (Figure 4). Gnathochilarium and labrum tightly appressed, mandibles not visible externally. Anterior margin of labrum smooth, with small depressed triangular area (Figs 4, 5A, B); without ramified cuticular papillae (as in *Brachycybe*). Paranota of ring five swept forward (anteriorly); distinctly bilobed in *corticarius* (Figure 2A). Paranotal caudolateral margins posterior to ring five sharply projecting caudally, unlike *Gosodesmus* where the paranotal margins are quadrate. Porosteles clearly differentiated, ozopores rimmed with peritrema, directed anteriorly on ring five (Figs 2, 5C), laterally on remaining rings. Telson: Hypoproct absent, preanal ring barrel-shaped (Figure 5D). Gonopods: Anterior gonopods rounded and robust; A6 spatulate (Figs 6–9) not with a distal series of thin ribbon-like styli, as in *Brachycybe*. Posterior gonopod long with P6 extended beyond A6, ending in a variable crown, which may be elongated, spatulate, or bifurcate (Figs 6–9) but never composed of a bundle of styli as in *Brachycybe*. P6 with medial spur-like process (possibly claw/tarsungulum). See also diagnosis of *Andrognathus* in Shear and Marek (2009).

######## Note.

*Andrognathus* was placed in Andrognathinae (a monotypic subfamily) due to the lobed condition of the fifth pair of paranota (Hoffman, 1980).

####### Andrognathus corticarius

Taxon classificationAnimaliaPlatydesmidaAndrognathidae

Cope, 1869

Figs 1 , 2 , 3 , 4 , 6 , 7 , 8 , 9 , Tables 1 , 2

Andrognathus corticarius Cope, 1869: 182; Gardner 1975: 15, figs 1, 5–7; Shear and Marek 2009: 157, figs 1–10. (There are no synonyms for A.corticarius.)

######## Type species.

Original type material lost. Collected by Cope “from Montgomery County, Virginia”.

######## Material examined.

Neotype: Male neotype (VTEC, MPE01942); 1 male from Virginia, Montgomery County, Christiansburg, Yellow Sulfur Springs Spa (37.1796, -80.3979, Elev. 607m), 30 June 2016 (Colls: P. Shorter, J. Means, V. Wong). Head and posterior body rings preserved as voucher. Collected on a hardwood log on a footpath between the old hotel gardens and an abandoned bowling alley.

Other material examined: 3 males, 3 females, details as for neotype; 109 males, 113 females, and 52 juveniles were examined from Virginia and nine other states (West Virginia, Tennessee, North Carolina, Florida, South Carolina, Alabama, Georgia, Ohio, and Kentucky), details in Suppl. material 1.

We here designate a neotype for *A.corticarius* because the holotype or syntypes have not been located and are presumed lost (see Material and methods section), and because our morphological studies indicate that “*A.corticarius*” may represent more than one species. To provide a basis for taxonomy of the group we have selected a specimen from the type locality which agrees in all details with the description given by Cope (1869).

######## Diagnosis.

Adult males of *A.corticarius* are distinct from other *Andrognathus* species based on the following combination of characters: Exoskeleton. Ring five with pleuroterga distinctly bilobed, papilioform (Figure 2A); lobe of anterior corner flared anteriorly, lobe of posterior corner bulging laterally, contrasting with the reduced lobes of *A.hoffmani* and *A.grubbsi* sp. n. Ring VI with metaterga singly lobed, with angulate paranota bearing a posterolaterally oriented ozopore, separating *A.corticarius* from *A.hoffmani* and *A.grubbsi* sp. n. in which the paranota are rounded and bear a laterally oriented ozopore (Figure 2). Paranota becoming progressively more directed posteriorly along subsequent body rings. Posterior gonopod with P6 terminating in a distally flared calyx, tip with many small serrations (Figs 6–8, Figure 9A), not spatulate as in *A.hoffmani* (Figure 9B) and *A.grubbsi* sp. n. (Figure 9C). Claw (ungulum) of P6 present, thorn-shaped, varying in size; claw not perpendicular to the shaft of the gonopod as in *A.grubbsi* sp. n.

######## Neotype details.

Body length (BL) = 17.9 mm, head width (HW) = 0.46 mm, collum width (CW) = 0.61 mm, metazonite width at 1/4 length of body (W1) = 0.81 mm, number of podous tergites (p) = 57, number of legs (l) = 210. NCBI accession # MH282831.

######## Variation.

*Andrognathuscorticarius* is known from the panhandle of Florida, north into southern Indiana and Pennsylvania (Figure 3). Variation in somatic characters of males and females is given in Table 1, and variation in gonopodal characters in Table 2. The morphology of the A6 and P6 podomeres and the claw of the P6 of the male gonopods of *A.corticarius* differed most across the distribution of the species (Table 2). Four individuals from the subset of geographically widespread samples differed from the neotype in two of these three characters of the male gonopods (Figs 6B, 7A, 8A, 8D); a male from Chesterfield County, South Carolina, deviates from the neotype in all three of these characters (Figure 8B).

**Table 2.** Variable gonopodal characters in *A.corticarius.* Neotype (*).

	A6 Ribbed	A6 Spatulate smooth curve	A6 Ribbed, scalloped edges	A6 Medial bifurcate hook	P6 claw Toothlike hook	P6 claw Bulbous, Rounded	P6 claw Pinched	P6 claw Bulbous, Rounded tooth lacking	P6 claw Bifurcate	Apex of P6 Singular fringed crown	Apex of P6 Bifurcate	Apex of P6 Bifurcate branched
MPE01998	+				+					+		
MPE01804		+					+		+			
MPE01942*		+			+					+		
MPE02169		+			+					+		
MPE01431		+				+			+			
AND043		+				+				+		
MPE01966			+		+					+		
AND040		+					+		+			
AND041		+					+		+			
AND039				+				+				+
AND005		+			+					+		
AND038		+							+		+	

######## Genetic analysis.

Genetic analysis using the COI region showed relatively low variation within eastern *A.corticarius* (mean pairwise distance = 0.16%). The aligned COI sequences were length invariant, trivial to align, and resulted in a matrix of 605 DNA base-pairs for 14 individuals. Individuals from Boone County, West Virginia and Scioto County, Ohio had a maximum pairwise distance of 0.99% (605 bp). The maximum pairwise distances between individuals from the same locality in Montgomery Co., Virginia, was 0.17% (605 bp).

######## Distribution and ecology.

**Figure 10.**
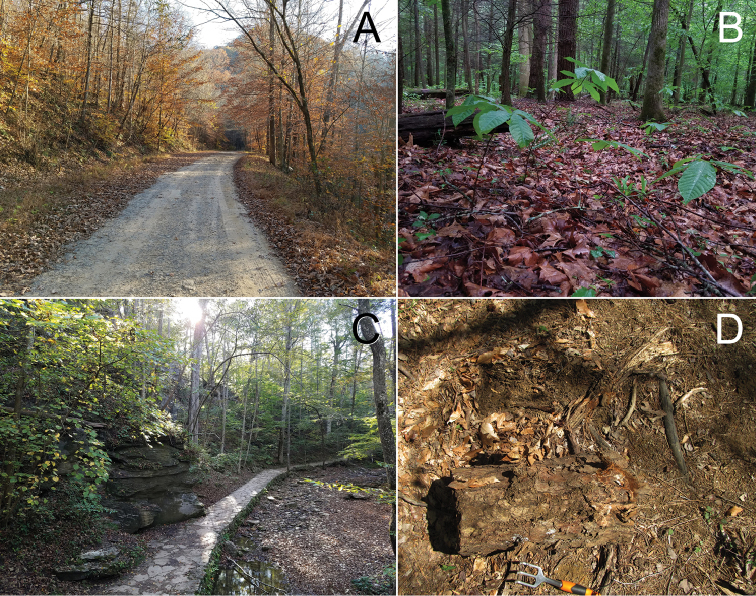
*Andrognathuscorticarius* habitat. **A** Boone County, West Virginia **B** Morgan County, Tennessee **C** Carter County, Kentucky **D** Montgomery County, Virginia.

*Andrognathuscorticarius* is now known from Indiana, Ohio, Pennsylvania, Kentucky, West Virginia, Virginia, Tennessee, North Carolina, South Carolina, Georgia, Alabama, and Florida (Figure 3). The map assembled here extended the known range of *A.corticarius* northward into southwestern Ohio and southeastern Pennsylvania and synthesized and expanded distributions from the literature (Figure 3). Based on the collection dates of museum specimens, the activity period of the species appears to span March – November, with the greatest number of specimens encountered mid-summer. Individuals are often found clinging to the underside of logs in mesic deciduous forests in aggregations of millipedes, including juveniles (Figure 1B). *Andrognathuscorticarius* is found from elevations of 51 m to 1160 m, and infrequently as lone adults in leaf litter and in drier habitats (Figure 10).

After some specimens of *A.corticarius* were collected in Pulaski Co., Virginia in 2016, a male and female were observed to be coiled together at the bottom of their container (Figure 1D). Upon closer inspection, the female was coiled around a cluster of eggs, with the male on top of her. The millipedes stayed in this configuration even when removed from their container and examined for approximately 20 minutes. This is a previously unobserved behavior in the genus, which was not known to demonstrate parental care.

####### Andrognathus grubbsi sp. n.

Taxon classificationAnimaliaPlatydesmidaAndrognathidae

http://zoobank.org/DCD92723-2649-41F6-89EB-41868CF48B99

Figs 2 , 5 , 9 , 11

######## Material examined.

Male holotype (VTEC, AND0044), 1 female paratype (VTEC, AND0051), 1 female paratype (VMNH, AND0045) from New Mexico, Catron Co., Gila National Forest, “near Glenwood”, 33.3254, -108.8847, uncertainty: 5000 m, August 1980 (collector: A. G. Grubbs).

######## Diagnosis.

Distinct from other members of the genus by the following: anterior lobe of pleurotergite V flared anteriorly (Figs 2C, 5C), but not distinctly bilobed, papilioform as in *A.corticarius* (Figure 2A). Apex of the P6 process bifurcate, each process flanged (Figure 9C), not a calyx as in *A.corticarius* (Figs 6–8, 9A). Distal process spatulate, bent at a 90° angle, covering the proximal process in a roof-like configuration (Figure 9C), contrasting with the upright, parallel processes of *A.hoffmani* (Figure 9B). Claw of the gonopod small, not large as in *A.corticarius* and *A.hoffmani*.

######## Description of male holotype.

**Figure 11.**
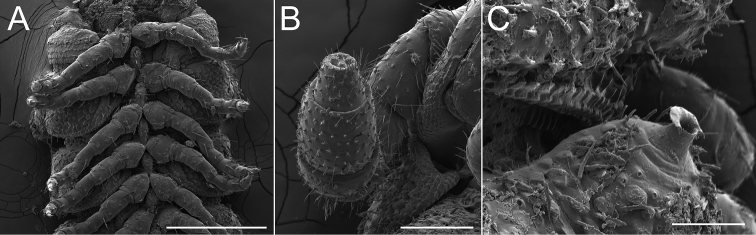
Somatic characters of *Andrognathusgrubbsi* sp. n. (catalog # AND0045). **A** Mid-body rings, ventral view **B** Distal antennomeres, ventral view **C** Ozopore of ring five, dorsal view. Scale bars: 0.3 mm (**A**), 0.1 mm (**B**), 0.05 mm (**C**).

Counts and measurements: number of podous tergites (p) = 45. Number of apodous tergites (a) = 2. Number of legs (l) = 170. (45 + 2 + T). Body length (BL) = 12.8 mm. Head width (HW) = 0.39 mm. Interantennal socket width (ISW) = 0.19 mm. Antennomere 6 width (AW) = 0.10 mm. Collum width (CW) = 0.45 mm. Metazonite width at 1/4 length of body (W1) = 0.69 mm. Metazonite length at 1/4 length of body (L1) = 0.23 mm. Metazonite height at 1/4 length of body (H1) = 0.45 mm. ***Body***: With characters from the genus diagnosis. Body with 47 rings, faded yellow in color due to long-term storage in ethanol. ***Head***: Pilose, pear shaped, anteriorly narrowed toward mouth. Eyeless. Antennae extending back to second body ring, relative antennomere lengths 6>2>5>3>4>7>1. Antennomeres short and globular, with obvious ~90° bend at fourth antennomere (Figure 11B). Head evident viewed dorsally, collum not covering head, anterior and posterior margins slightly sinuate medially. ***Exoskeleton***: Prozonites and metazonites of rings 2–4 distinct in appearance; paranota arising from anterior portion of ring and lacking ozopores (Figure 5C). Fifth ring noticeably elongated, metazonite bearing a distinct lateral and posterior ridge, and transverse groove (Figure 5C). Paranota of the fifth ring lobed, lacking bifurcation and posterior placement (Figs 2C, 5C). Ozopores beginning on the fifth ring, oriented anterolaterally; on following rings oriented posterolaterally. Porosteles elongated, (in contrast to short porosteles of other members of the genus), with a doughnut-like rim (Figure 11C). Ring VI and other ozoporiferous rings subequal in length, paranota directed laterally, peritremata directed posterolaterally. Posterior-most paranota and peritremata gradually shifting in orientation; directed posteriorly, with sharp caudolateral corners. Prozonites lacking setae, covered with minute disc-like tubercles (Figs 5C, 11A). Metazonites setose, with fewer tubercles limited to median transverse groove and anterior portion of paranota. Metazonal limbus lined with uniform rectangular tabs (Figs 5C, 11A). Pleurites covered with disc-like tubercles and setae, pleurites of apodous rings overlapping (Figure 5D). Sternites with bulbous median knob with a few setae and raised tubercles (Figure 11A). ***Legs***: Podomeres of walking legs bearing long setae. Coxa globular, with medial excavation, anterior face with raised tubercles. Coxae contiguous anteromedially (Figure 11A). Trochanters thin, other podomeres rectangular with slight widening at apex. Prefemur longest podomere, following podomeres subequal. Tarsal claw simple, slightly curved (Figure 11A). Single comb row of stout setae on anteromedian edge of leg pairs one through three. ***Gonopods***: Ninth and tenth leg pairs modified into simple leg-like gonopods (Figure 9C). Anterior gonopod with stout coxae (A1) bearing medium triangular coxal apophyses that project anteriorly (Figure 9C). Subsequent podomeres (A3 – A6) wider than long. Sixth podomere (A6) spade-shaped, medially spatulate, with setae (Figure 9C). Posterior gonopod narrower than anterior (Figure 6C). Coxae and second podomeres (P1, P2) stout, slightly wider than long. Subsequent podomeres (P3 – P5) tapering to an elongated P6 that meets the apex of the anterior gonopods. Claw (ungulus) of P6 a stout isosceles triangle on medial side (Figure 9C). P6 bifurcate distally, with two processes (Figure 9C). Proximal process flange-like, projecting upward. Distal process spatulate, twisting over the proximal process at a 90° angle (Figure 9C). Color: Faded yellow after being stored in alcohol for 37 years.

######## Paratypes.

(female) AND051 and AND0045. Counts and measurements: p = 53 and 57, a = 2, l = 194 and 210, BL = 19.40 mm and 17.00 mm, CW = missing and 0.46 mm, W1 = 0.83 mm and 0.90 mm. Somatic characters similar to holotype.

######## Etymology.

This species is named for its collector, cave biologist Andy Grubbs. While not a myriapodologist, his collecting philosophy was that someday there would be someone interested in the specimens that he collected. Now, almost 40 years later, that philosophy has been vindicated. The specific name is a genitive noun derived from his surname.

######## Distribution and ecology.

*Andrognathusgrubbsi* sp. n. is known only from the type locality in “Glenwood, New Mexico”. The exact location is unknown; however, proximity of Glenwood to the U.S. Forest Service Bighorn Campground in Gila National Forest suggests that the specimens may have been collected there. The campground site is situated in the Arizona/New Mexico Mountains ecoregion (Level III), likely in the Madrean Lower Montane Woodlands (Level IV) (Omernik 1987). The dominant vegetation in the area is composed of pinyon pines and juniper trees (Gila National Forest 2018). This type locality is considerably separated from other known *Andrognathus* localities. *Andrognathushoffmani* in Mexico is ca. 1270 km southeast of the specimens from New Mexico, and *A.corticarius* in Alabama, the westernmost record for the species, is ca. 1990 km southeast.

## Discussion

The genus *Andrognathus* currently comprises three species: *A.corticarius*, *A.hoffmani*, and *A.grubbsi* sp. n. *Andrognathusgrubbsi* sp. n. falls unambiguously within the genus (based on the modified fifth ring), but is geographically and morphologically distinct from its congeners. The possibility that there are cryptic species within *A.corticarius* was first suggested based on a limited sample of individuals, and without molecular phylogenetic data (Shear and Marek 2009). Our investigation with scanning electron microscopy within a geographical context inferred with a larger sample of natural history specimens has provided evidence that *A.corticarius* may encompass several distinct species. We base this hypothesis on our observations of gonopod variation across the distribution of *A.corticarius* (Figs 6–8). Northern and eastern individuals tend to vary little from the type, and differentiation appears to be more substantial further south. The claw of P6 tends to become more complex in the southern part of the range: appearing either bifurcate or bulbous. The apex is also bifurcate in many of the southern individuals. The exception to this trend is an individual (AND0039) from South Carolina (Figure 8B), which is an outlier to distributional trends and is highly divergent morphologically from the type. This anatomical variation in *A.corticarius* gonopods across its wide distribution in the eastern U.S. is noteworthy when compared to the many millipede species with large distributions and conserved gonopod shape. For example, *Pleurolomaflavipes* Rafinesque, 1820 and *Gyalostethusmonticolens* (Chamberlin, 1951) (Polydesmida: Xystodesmidae) both have gonopods that vary only slightly across their > 3 million km^2^ and > 300,000 km^2^ geographical distributions (Hoffman 1960, 1965, Shelley 1980). The confamilial species *Brachycybelecontii* (Wood, 1864) possesses no readily apparent gonopodal differences across its distribution (> 450,000 km^2^) in the eastern U.S. (Brewer et al. 2012), a distribution which is largely congruent with that of *A.corticarius*. However, in *B.lecontii* distinct genetic differences are observed in nuclear (192fin gene region) and mitochondrial (COI) DNA that indicates that at least four geographically separated and non-overlapping clades exist (Brewer et al. 2012). In contrast, in *A.corticarius*, we found the opposite and observed noticeable gonopodal differences between geographically widespread populations and yet a paucity of genetic differences in our COI data. Due to a lack of congruence between our morphological and genetic datasets, and lack of geographical consistency in gonopodal shape variation, we refrain from formally naming these distinct populations as species. In future investigations of species boundaries in *A.corticarius*, an integrative approach implementing additional genetic loci, ecological niche data, and a detailed analysis of gonopod morphology should be used.

It is uncertain if populations of *A.corticarius* vary in other gene loci along its distribution. Though it is uncommon for the COI gene region to be nearly invariant, similar results have been seen in other dispersal-limited invertebrate taxa. This low genetic variation across geographically separated populations is surprising given expectations of rapid evolution in animal mitochondrial loci due to the haploid nature of COI and its maternal mode of inheritance (Ladoukakis and Zouros 2017). In a study of the harvestman genus *Acuclavella* Shear, 1986, sampling of five widely-separated species in the Pacific Northwest and COI sequencing indicated a lower than expected within-species nucleotide variation (Richart and Hedin 2013) similar to our COI variation in *A.corticarius*. In future work, other genes should be targeted and utilized in analyzing *A.corticarius* evolution to investigate genetic differences between localities and to reconcile differing gene histories that may be more informative than just a single genealogy.

By assembling an updated distribution map of *Andrognathus*, we have shown that the genus occurs in the eastern U.S. (in the Appalachian Mountains and Coastal Plain), and in two isolated locations in Glenwood, New Mexico and Cerro el Potosi, Mexico. Other millipede taxa with a similar distribution are *Rhysodesmus* Cook, 1895, which has two species in the eastern U.S. and nearly 70 known in Texas and Mexico (Marek et al. 2014). Similar distributions occur in the fern species *Pellaeawrightiana* Hooker, 1858 (http://efloras.org/object_page.aspx?object_id=5145&flora_id=1; accessed July 30, 2018) and *Aspleniumseptentrionale* (Linneaus, 1758) (http://efloras.org/object_page.aspx?object_id=5187&flora_id=1; accessed July 30, 2018) The mountains of southern New Mexico and Texas, and the Sierra Madre in Mexico likely hold additional millipede taxa. Searching these locations for additional species of *Andrognathus* would be fruitful in understanding diversity of the genus and evolution of the taxon.

*Andrognathus* species occur in mesic forested habitats at mid-elevations (51–1523 m a.s.l.). As global warming affects the distribution of species and causes aridification of habitats, *Andrognathus* millipedes may face a limited set of options. These invertebrates may shift to higher elevations or north facing slopes to avoid desiccation. By understanding the alpha-taxonomy and distribution of these dispersal limited mesic-adapted species today, conservation specialists will have the baseline data relevant to the conservation needs of these arthropods, and relatives.

## Supplementary Material

XML Treatment for Andrognathus

XML Treatment for Andrognathus corticarius

XML Treatment for Andrognathus grubbsi

## Supplementary materials

Supplementary material 1 Specimen catalog Data type: specimens data This is an open access article distributed under the terms of the Creative Commons Attribution License (CC BY 4.0), which permits unrestricted use, distribution, and reproduction in any medium, provided the original author and source are credited.Patricia L. Shorter, Derek A. Hennen, Paul E. Marek

Supplementary material 2 Locality data Data type: occurence This is an open access article distributed under the terms of the Creative Commons Attribution License (CC BY 4.0), which permits unrestricted use, distribution, and reproduction in any medium, provided the original author and source are credited.Patricia L. Shorter, Derek A. Hennen, Paul E. Marek

Supplementary material 3 Mean uncorrected pairwise distance matrix Data type: statistical data This is an open access article distributed under the terms of the Creative Commons Attribution License (CC BY 4.0), which permits unrestricted use, distribution, and reproduction in any medium, provided the original author and source are credited.Patricia L. Shorter, Derek A. Hennen, Paul E. Marek

## References

[B1] Attems C (1951) Revision systématique des Colobognatha (Myriapodes Diplopodes) et description d’espèces nouvelles. Mémoires du Muséum national d’Histoire naturelle, Paris (n.s. A) 3: 193–231.

[B2] Brewer MS Spruill CL Rao NS Bond JE (2012) Phylogenetics of the millipede genus *Brachycybe* Wood, 1864 (Diplopoda: Platydesmida: Andrognathidae): Patterns of deep evolutionary history and recent speciation. Molecular Phylogenetics and Evolution 64: 232–242. 10.1016/j.ympev.2012.04.003 22516430

[B3] BugGuide.net (2016) BugGuide.net: Identification, Images, & Information for Insects, Spiders & their Kin for the United States & Canada. http://bugguide.net/node/view/1136695

[B4] Cook OF Loomis HF (1928) Millipeds of the order Colobognatha with descriptions of six new genera and type species, from Arizona and California. Proceedings of the U.S. National Museum 72(18): 1–28. 10.5479/si.00963801.72-2714.1

[B5] Cope ED (1869) Synopsis of the extinct Mammalia of the cave formations in the United States, with observations on some Myriapoda found in and near the same, and on some extinct mammals of the caves of Anguilla, WI, and of other localities. Proceedings of the American Philosophical Society 11(81): 147–192. http://biodiversitylibrary.org/page/31205032

[B6] Enghoff H Dohle W Blower JG (1993) Anamorphosis in millipedes (Diplopoda)–the present state of knowledge with some developmental and phylogenetic considerations. Zoological Journal of the Linnean Society 109(2): 103–234. 10.1111/j.1096-3642.1993.tb00305.x

[B7] Enghoff H Golovatch S Short M Stoev P Wesener T (2015) Diplopoda–taxonomic overview. In: Minelli A (Ed.) Treatise on Zoology – Anatomy, Taxonomy, Biology.The Myriapoda 2. Brill, Leiden, 363–454. 10.1163/9789004188273_017

[B8] Ewing B Hillier L Wendl MC Green P (1998) Base-calling of automated sequencer traces using Phred. I. Accuracy assessment. Genome Research 8: 175–185. 10.1101/gr.8.3.175 9521921

[B9] Folmer O Black M Hoeh W Lutz R Vrijenhoek R (1994) DNA primers for amplification of mitochondrial cytochrome c oxidase subunit I from diverse metazoan invertebrates. Molecular Marine Biology and Biotechnology 3(5): 294–299. http://www.mbari.org/wp-content/uploads/2016/01/Folmer_94MMBB.pdf 7881515

[B10] Gardner MR (1975) Revision of the millipede family Andrognathidae in the Nearctic region. Memoirs of the Pacific Coast Entomological Society 5: 1–61.

[B11] Gila National Forest (2018) Gila National Forest, United States Department of Agriculture, Forest Service. https://www.fs.usda.gov/recarea/gila/recarea/?recid=1969 [29 April 2018]

[B12] Golovatch S Evsyukov A Reip HS (2015) Colobognatha millipedes in the Caucasus (Diplopoda: Polyzoniida, Platydesmida, Siphonocryptida). Zootaxa 3972(2): 250–266. 10.11646/zootaxa.3972.2.6. 26249491

[B13] Hebert PD Cywinska A Ball SL (2003) Biological identifications through DNA barcodes. Proceedings of the Royal Society of London B: Biological Sciences 270: 313–321. 10.1098/rspb.2002.2218 PMC1691236 12614582

[B14] Hoffman RL (1960) Revision of the milliped genus *Cherokia* (Polydesmida: Xystodesmidae). Proceedings of the United States National Museum 112(3436): 227–264 10.5479/si.00963801.112-3436.227

[B15] Hoffman RL (1965) Proceedings of the United States National Museum 117: 305–347. 10.5479/si.00963801.117-3514.305

[B16] Hoffman RL (1980 [1979]) Classification of the Diplopoda. Muséum d’Histoire naturelle, Genève, 237 pp. [date of publication 3 June 1980]

[B17] Hoffman RL (1999) Checklist of millipeds of North and Middle America. Virginia Museum of Natural History Special Publications, Martinsville, 584 pp https://www.fieldmuseum.org/sites/default/files/hoffman_checklist_1999.pdf

[B18] Ladoukakis ED Zouros E (2017) Evolution and inheritance of animal mitochondrial DNA: rules and exceptions. Journal of Biological Research-Thessaloniki 24: 2. 10.1186/s40709-017-0060-4 PMC5282644 28164041

[B19] Latzel R (1884) Note sur les Julides de la Belgique, suivie da la description d’une espèce nouvelle. Extrait des Comptes rendus de la Société Entomologique de Belgique. https://babel.hathitrust.org/cgi/pt?id=hvd.32044107192346.

[B20] Lewis JGE (1984) Notes on the biology of some common millipedes of the Gunung Mulu National Park, Sarawak, Borneo. Sarawak Museum Journal 33(54): 179–185.

[B21] Maddison WP Maddison DR (2010) Mesquite: a molecular system for evolutionary analysis. Version 2.74. http://mesquiteproject.org/

[B22] Marek PE Shear WA Bond JE (2012) A redescription of the leggiest animal, the millipede *Illacmeplenipes* with notes on its natural history and biogeography (Diplopoda, Siphonophorida, Siphonorhinidae). ZooKeys 241: 77–112. 10.3897/zookeys.241.3831 PMC3559107 23372415

[B23] Marek PE Tanabe T Sierwald P (2014) A species catalog of the millipede family Xystodesmidae (Diplopoda: Polydesmida). Virginia Museum of Natural History Publications 17: 1–117. ttp://www.vmnh.net/content/File/Research_and_Collections/VMNHSpecialPub17.pdf

[B24] MCZBase (2017) MCZBase: The Database of Zoological Collections, Museum of Comparative Biology-Harvard University. http://mczbase.mcz.harvard.edu/SpecimenSearch.cfm.

[B25] Means JC Francis EA Lane AA Marek PE (2015) A general methodology for collecting and preserving xystodesmid and other large millipedes for biodiversity research. Biodiversity Data Journal 3(3): e5665. 10.3897/BDJ.3.e5665 PMC4563156 26379461

[B26] Omernik JM (1987) Ecoregions of the conterminous United States. Annals of the Association of American Geographers 77: 118–125. 10.1111/j.1467-8306.1987.tb00149.x

[B27] Read HJ Enghoff H (2009) The order Siphonophorida – A taxonomist’s nightmare? Lessons from a Brazilian collection. Soil Organisms 81(3): 543–556. http://www.senckenberg.de/files/content/forschung/publikationen/soilorganisms/volume_81_3/22_read.pdf

[B28] Richart CH Hedin M (2013) Three new species in the harvestmen genus *Acuclavella* (Opiliones, Dyspnoi, Ischyropsalidoidea), including description of male *Acuclavellaquattuor* Shear, 1986. ZooKeys 311: 19–68. 10.3897/zookeys.311.2920 PMC3698555 23825441

[B29] Rios NE Bart HL (2010) GEOLocate (Version 3.22)[computer software]. Tulane University Museum of Natural History, Belle Chasse, LA.

[B30] Rodriguez J Jones TH Sierwald P Marek PE Shear WA Brewer MS Kocot KM Bond JE (2018) Step-wise evolution of complex chemical defenses in millipedes: a phylogenomic approach. Scientific Reports 8(1): 3209. 10.1038/s41598-018-19996-6 PMC5816663 29453332

[B31] Shear WA Marek PE (2009) *Andrognathushoffmani*, n. sp., a second species in the genus and the first species of Andrognathidae from México (Diplopoda, Platydesmida, Andrognathidae). Festschrift in honor of Richard Hoffman, Memoirs of the Virginia Museum of Natural History 16: 149–158. https://www.fieldmuseum.org/sites/default/files/shear%26marek_festschrift_2009.pdf

[B32] Shear WA (2015) The chemical defenses of millipedes (Diplopoda): biochemistry, physiology and ecology. Biochemical Systematics and Ecology 61: 78–117. 10.1016/j.bse.2015.04.033

[B33] Shelley RM (1978) Millipeds of the eastern Piedmont region of North Carolina, U.S.A. (Diplopoda). Journal of Natural History 12: 27–79. 10.1080/00222937800770051a

[B34] Shelley RM (1980) Revision of the milliped genus *Pleuroloma* (Polydesmida: Xystodesmidae). Canadian Journal of Zoology 58: 129–168. 10.1139/z80-017

[B35] Shelley RM (2000) Annotated checklist of the millipeds of North Carolina (Arthropoda: Diplopoda), with remarks on the genus *Sigmoria* Chamberlin (Polydesmida: Xystodesmidae). The Journal of the Elisha Mitchell Scientific Society 116(3): 177–205. https://dc.lib.unc.edu/cdm/singleitem/collection/jncas/id/3612/rec/2

[B36] Shorthouse DP (2010) SimpleMappr, an online tool to produce publication-quality point maps. http://www.simplemappr.net.

